# Intraoral excision of a huge retropharyngeal lipoma causing dysphagia and obstructive sleep apnea^☆^

**DOI:** 10.1016/j.bjorl.2016.10.011

**Published:** 2016-11-17

**Authors:** Umit Aydin, Omer Karakoc, Murat Binar, Fatih Arslan, Mustafa Gerek

**Affiliations:** a Gulhane Military Medical Academy, Department of Otolaryngology, Head and Neck Surgery, Ankara, Turkey; b Ankara Mevki Military Hospital, Department of Otolaryngology, Head and Neck Surgery, Ankara, Turkey

## Introduction

Lipomas, derived from the mesenchyme, are the most common soft-tissue tumors in the body, but only 15% of them are located in the head and neck region.^1^ Head and neck lipomas usually arise from posterior cervical triangle and the incidence of lipoma in the retropharyngeal region is very rare.^2^, ^3^ Herein, we present a case of retropharyngeal lipoma causing progressive dysphagia and obstructive sleep apnea (OSA) which is treated by transoral surgical excision. We also review the literature to discuss the management of retropharyngeal lipomas causing OSA. To the best of our knowledge, present case shows the most huge retropharyngeal lipoma in the English literature causing dysphagia and OSA.

## Case report

**Figure 1:**
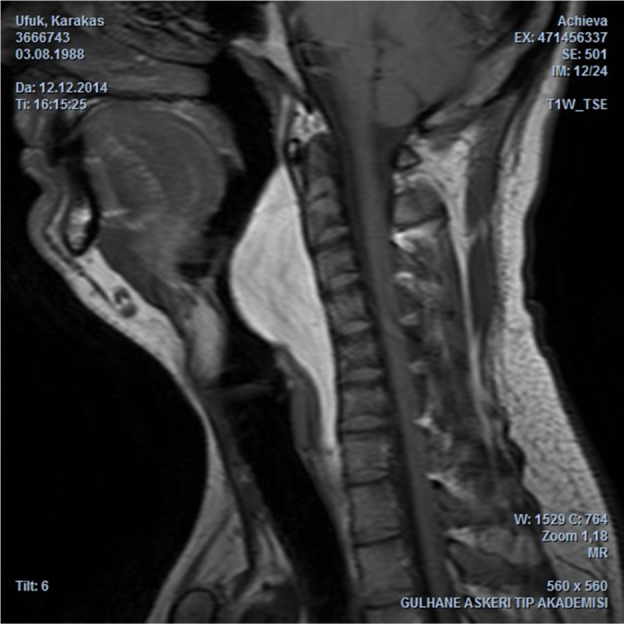
Sagittal T1-weighted MRI depicts the hyperintense retropharyngeal mass lesion similar to fat.

**Figure 2:**
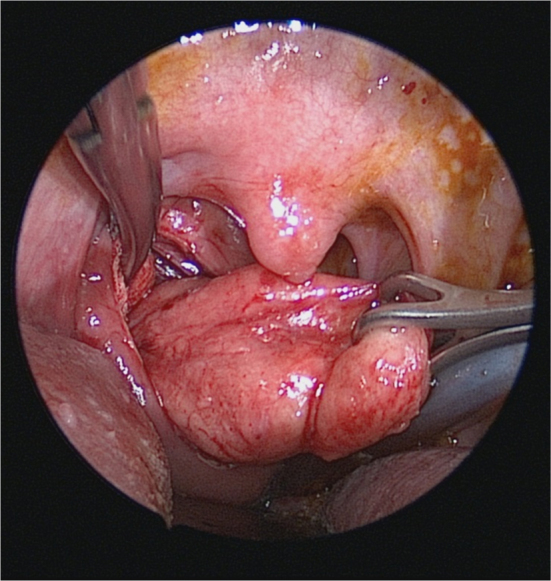
Dissection of the mass in the retropharyngeal space.

**Figure 3:**
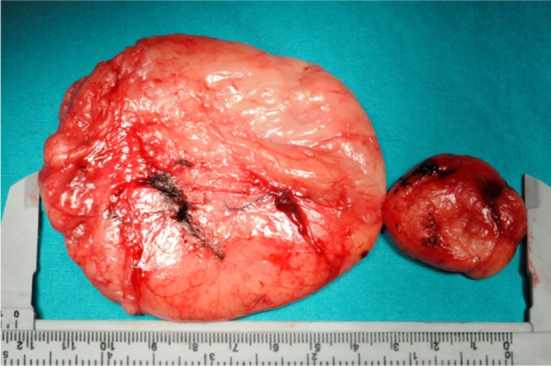
Gross specimen photograph of the retrophayngeal lipoma.

**Figure 4:**
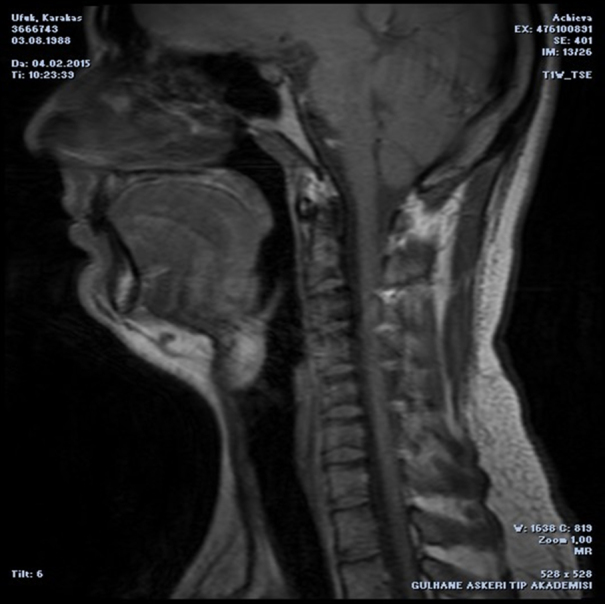
Postoperative sagittal T1-weighted MRI showed that the mass was totally resected.

A 24 year-old man presented with a one year history of progressive snoring, excessive daytime sleepness, dyspnea and dysphagia to solid materials. The examination revealed posterior pharyngeal wall bulging extending from oropharynx to hypopharynx. In endoscopic evaluation, the epiglottis was touching the mass lesion and it was not possible to see the vocal cords. The mucosa was intact and smoothless. The polysomnography identified a severe OSA with an Apnea-Hypopnea Index (AHI) of 96.8. Computed Tomography (CT) revealed a homogeneous retropharyngeal mass extending from level C2 to C6. In the Magnetic Resonance Imaging (MRI), the mass lesion was observed to be originated from posterior prevertebral region in the retropharyngeal space with high signals in T1 (Fig. 1) and T2 images, as well as high signal attenuated with the fat supression and no relationship with bone. These findings suggested that the retropharyngeal mass lesion was of lipomatous origin. The transoral surgical excision under general anesthesia was advised to the patient. After orotracheal intubation, a mouth gag is placed. Vertical incision was performed on the posterior pharyngeal mucosa with a unipolar coutery and the superior constrictor muscle and deep cervical fascia were dissected, followed by the blunt mass dissection (Fig. 2), and then the retropharyngeal mass was completely removed. Macroscopically, the tumor was yellowish, bilobed, well encapsulated, and measured approximately 12 × 7 cm (Fig. 3). The postoperative period was uneventful. Tracheotomy was not needed. A nasogastric tube was sustained for 5 days during postoperative period and the patient was given one-week antibiotic treatment. The histopathologic diagnosis of the lesion was lipoma. At the end of the second month, the AHI, the minimum O_2_ saturation, and the percentage of sleep time with O_2_ saturation below 90% improved from 96.8 to 10, from 61% to 87%, and from 54.8% to 11.4%, respectively. Control MRI revealed no residue of the lipoma (Fig. 4) and the patient was completely relieved from the complaints following the excision.

## Discussion

The retropharyngeal area is a potential space located between buccopharyngeal fascia anteriorly, and prevertebral fascia posteriorly. This space extends from the base of the skull to the mediastinum. Lymph nodes, nerves, and fat are the essential components of this space.^4^ Tumors of the retropharyngeal space are relatively uncommon. Lipoma is one of these tumors.^5^

Lipomas are slow-growing benign mesenchymal tumors that arise from the adipose tissue and consist of mature adiposits. Head and neck lipomas usually located at subcutaneus region of the neck.^6^ Submucosal and deep situated lipomas such as nasopharynx, oropharynx and parapharyngeal region are infrequent. Lipoma is seen as homogenous, hipodense, well-defined non-enhancing lesion on CT. Homogeneous fatty attenuation upon CT has been indicated for lipomas. While CT is useful for diagnosing lipoma, MRI is preferred over CT due to clear and detailed images of soft tissue.^7^ MRI is also important for delineating the limits and soft issue extension of lesion.

Retropharyngeal lipomas may grow to considerable size before becoming symptomatic. Common symptoms are dysphagia, dyspnea and snoring related to airway obstruction.^3^ OSA may be coincide with airway obstruction.^8^ In OSA, repeated episodes of partial or complete upper airway obstruction that causes apnea, hypopnea, desaturation and sleep fragmentation lead to chronic hypoxia. It can occur due to many pathophysiological factors such as neuromuscular dysfunction, anatomic abnormalities, etc.^3^ Severe obstruction of the upper airway due to the lipoma was the essential cause of OSA in our case. Retropharyngeal lipomas are relatively rare in the etiology of OSA. In the literature review we found four cases of retropharyngeal lipoma regarding polysomnography-proven OSA^2^, ^3^, ^9^, ^10^ (Table 1).

**Table 1:** The cases of retropharyngeal lipomas related with polysomnography-proven OSA.

Case n°	Sex	Age	Treatment	Size (cm)	Pre-treatment AHI	Post-treatment AHI	References
1	F	11	Transoral excision	8 × 4	13	–	Gong
2	F	44	CPAP	5 × 4	38	–	Tuncyurek
3	F	73	Transoral excision	8 × 4	43	12	Piccin
4	M	40	Transcervical excision	11 × 7	34	–	Namyslowski
5	M	24	Transoral excision	12 × 7	96.8	10	Present case

Complete surgical removal is the first choice of treatment. The surgical approach changes depending on the location of lipoma. Transoral excision is most preferred way for the surgery even in the huge lipomas of retropharyngeal region. This is possible because lipomas are usually well-encapsulated.^3^ Transoral excision has lower postoperative morbidity compared to transcervical approach, but in case of prominent parapharyngeal extension, transcervical approach may be preferred.^7^, ^8^ Continuous Positive Airway Pressure (CPAP) may be an option for the elderly patients with high comorbidity.

## Conclusion

The resolution of dysphagia and improvement in the polysomnography findings following the operation approved the obstructive character of the lesion. Upper airway examination should be carefully performed for patients with dysphagia and sleep apnea to exclude the retropharyngeal lesions such as lipomas. Transoral approach was the best choice for the surgical excision in our case.

## Conflicts of interest

The authors declare no conflicts of interest.
